# Buckling Analysis of Pultruded Glass Fiber Reinforced Polymer (GFRP) Angle Sections

**DOI:** 10.3390/polym12112532

**Published:** 2020-10-29

**Authors:** Rahima Shabeen Sirajudeen, Rajesh Sekar

**Affiliations:** College of Engineering Guindy, Anna University, Tamil Nadu 600025, India; rajeshsekar91@gmail.com

**Keywords:** pultruded glass-fiber reinforced polymer, angle columns, axial compression, buckling

## Abstract

Glass fiber reinforced polymers (GFRP), with their advantage of corrosion resistance, have potential to be used as structural members in civil engineering constructions. Pultruded GFRP angle section trusses could be used instead of steel sections in remote areas and in areas prone to corrosion. The objective of this paper is to study the strength of GFRP angle sections under concentric axial load. Glass fiber reinforced polymer (GFRP) made of E-glass and Isophthalic polyester resin and manufactured by pultrusion process was used for the experimental study. Two GFRP angle sections of size 50 × 50 × 6 mm and 50 × 50 × 4 mm and lengths 500 mm, 750 mm, and 1000 mm were chosen for the study. Further, finite experimental element analysis of the GFRP angle sections was done using ANSYS software and validated with the experimental results. The validated FE model was used for parametric studies varying the slenderness ratio and flange width to thickness ratio (b/t) ratio. It was observed that length of the specimen and thickness influenced the buckling load and buckling mode. An increase in *b*/*t* ratio from 8.3 to 12.5 decreases the load carrying capacity by almost 60% at a slenderness ratio of 50.

## 1. Introduction

Use of fiber reinforced polymer (FRP) in civil structural applications has been constantly increasing owing to its advantageous properties of greater corrosion resistance, low maintenance requirement and high durability even in aggressive environments when compared to the traditional construction materials like steel and reinforced concrete. FRP have potential to be used as an alternative construction material in offshore constructions, chemical plants, and transmission line towers. However, the FRP are also heterogenous, orthotropic, brittle, and not ductile when compared to steel which is homogeneous, isotropic, and ductile. Hence, the well-established theories and concepts used for design of structural steel elements cannot be used as such for FRP member. There is a need for a thorough investigation on the behavior of a FRP member to derive reliable design equations.

Angle sections are typically used as truss members in towers and are subjected to axial tension or compression and are traditionally made of steel. Fewer studies are available on GFRP angle section as compared to studies on GFRP I sections [1] and there is a lack of reliable design standards on angle sections for design engineers [2].

Many researchers have studied the behavior pultruded FRP sections of different shapes namely angle shaped sections [2,3,4], I shaped sections [5,6,7,8,9,10,11,12,13], Channel [14,15], box [16,17], and tubular sections [18]. Barbero and Tombli [5,6] investigated critical load of long GFRP I-section columns. A simplified method based on Southwell’s method based on Eulers buckling was used to determine the critical buckling load. Hashem and Yuan [16] conducted axial compressive strength studies on GFRP composite universal and box section columns manufactured by pultrusion process using E-glass fibers and polyester and vinylester resins. Euler’ s formula was employed to obtain critical loads for the slender columns. The classical plate theory was used to predict buckling load of short columns. Based on experimental evaluations and analytical results, a slenderness-ratio-based criterion was established for distinguishing between short and long composite column behaviors. Cardoso et al. [17] developed compressive strength equations for box columns considering global slenderness, plate slenderness, and imperfection factor. Laudiero et al. [19] investigated the sensitivity of initial imperfections for wide and narrow flange I-section columns under pure compression. Cintra et al. [20] studied the performance and strength of pultruded GFRP stub columns subjected to short term concentric loading.

Research papers are also available on the application of FRP pultruded sections in transmission line towers. Godat et al. [1] investigated the replacement of traditional materials (steel, wood, and concrete) in electricity transmission lines by fiber glass pultruded members. Various cross-sections made of E-glass and either polyester or vinylester matrix were tested. Angle-section, square-section, and rectangular-section specimens were subjected to axial compression and I-section and W-section specimens were tested under bending. The experimental results were summarized in terms of the failure mode, critical buckling load, and load–displacement relationships. Design equations available in FRP design manuals and analytical methods proposed in the literature were used to predict the critical buckling load. Selvaraj et al. [21] successfully tested a 66 kV transmission line tower made with GFRP composite angle sections. Selvaraj [22] conducted experiments on X-braced panel of transmission line tower made from FRP pultruded sections. Mathematical model of individual members and members in the X-braced panel were generated using FEM software. Prasad Rao et al. [2] investigated the failure of full-scale 24 m triangular lattice GFRP communication tower. Experimental, analytical and numerical studies were conducted on GFRP angles subjected to axial compression. Further the behavior of GFRP 60° and 90° angles in lattice system were studied and it was observed that the reason for failure of the 60° angle tower was the flexural-torsional buckling of leg member. Balagopal et al. [23] carried out experimental investigation on X-braced Transmission line tower substructure and observed that the tower failed prematurely due to the excessive bending stress exerted on the free length of stub member.

It is observed from literature that buckling is the dominant mode of failure in the experimental studies as opposed to crushing failure because of the reduced modulus of GFRP members. Buckling observed was either flexural buckling, flexural-torsional buckling, or local buckling depending on the shape, geometry, and length of the section. Theoretical studies have also been done to establish equations to predict the buckling loads of these members based on Euler global buckling equations and plate buckling equations for flexural, flexural-torsional buckling and local buckling.

In the present study, buckling analysis of pultruded GFRP angle sections subjected to concentric axial load is carried out using Finite element analysis software ANSYS. The scope of the study includes: (i) Experimental study on GFRP angle section of different slenderness ratio and width-to-thickness ratio; (ii) developing a finite element model in ANSYS; (iii) validation of finite element analysis results with experimental results of [3] and with the results of the experimental study carried out at College of Engineering Guindy, Anna University; and (iv) conducting a parametric study using the validated Finite Element Model (FEM) varying the slenderness and width-to-thickness ratio of the GFRP angle section.

## 2. Finite Element Analysis

**Figure 1:**
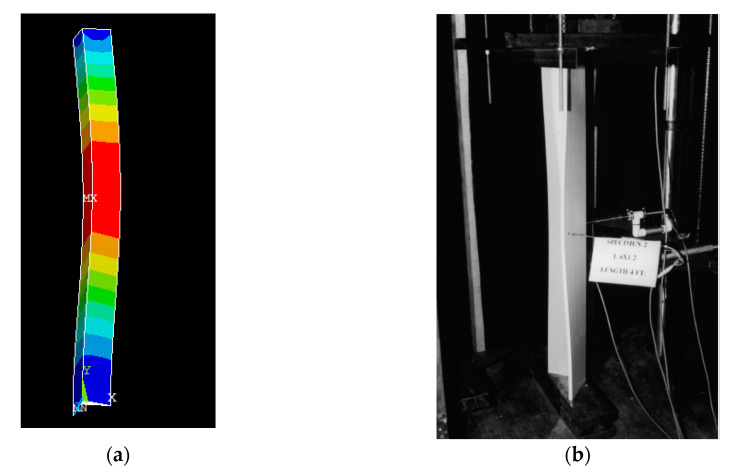
Buckling mode shape validation (**a**) Finite Element Analysis; (**b**) experiment Zureick and Steffen [3].

Numerical study on the buckling behavior of angle section was done using Finite Element Method (FEM). The angle sections were modeled by using ANSYS FE software and compared with the experimental results of [3]. The angle section was of size 102 × 102 × 12.7 mm (b × b × t) and length 1524 mm. GFRP material was assumed to be linear, elastic and orthotropic in the FE model. The material properties of the section are longitudinal modulus (E_L_) = 20.8 GPa, In-plane shear modulus (G_L_) = 3.3 GPa, longitudinal strength (F_LC_) = 308 MPa and In-plane shear strength = 68 MPa. “SHELL181” quadrilateral element was used to model the GFRP angle sections. “SHELL 181” is a four-noded element with six degrees of freedom at each node. It has linear/nonlinear and large rotation capabilities and can be used to model composite materials. The geometry of the angle section was automatically meshed by using mapped meshing algorithm. The end constrains at the extremities were modelled to represent simply supported boundary conditions. The bottom extremity of the angle section was restrained against all translational motions and the top extremity was also restrained against all translational motion except in the longitudinal direction. Material failure criteria was not specified as material crushing was a not predominant mode of failure and this study focusses on buckling behavior of the sections. A vertical load of unit total magnitude was applied at the nodes at the extremity of the column. The eigenvalue solution was obtained using the block Lanczos algorithm in ANSYS software. Buckling load and buckling mode shape of the angle section obtained from finite element analysis are compared with experimental results of Zureick and Steffen [3]. Table 1 shows the Finite Element Analysis (FEA) and experiment [3] results. Figure 1 shows the buckled mode shape of FEA and experiment [3]. It is observed from Figure 1 and Table 1 that the FEA is in good correspondence with experiment results [3].

## 3. Experimental Program

**Figure 2:**
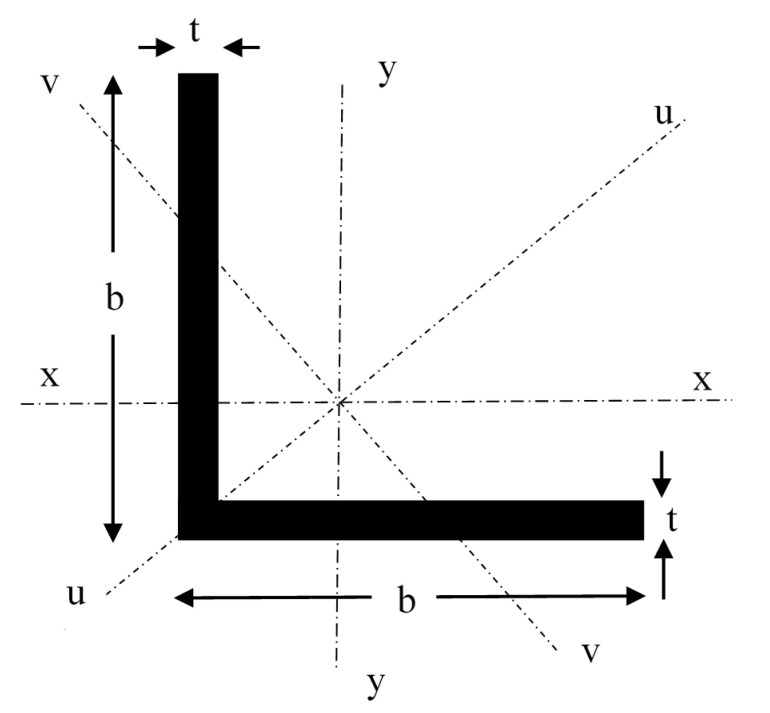
Geometry of angle section.

Two sizes of angle sections namely 50 × 50 × 4 mm (b × b × t) and 50 × 50 × 6 mm (b × b × t) and lengths 1000 mm, 750 mm, and 500 mm were chosen for the experimental study based on sections available in the market. Figure 2 shows the cross-section of a typical angle section.

**Figure 3:**
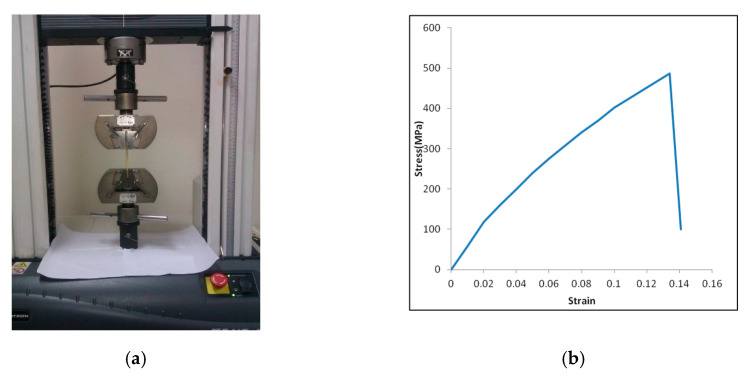
(**a**) Tensile test of GFRP coupon; (**b**) stress–strain curve of GFRP.

GFRP angle sections were manufactured by pultrusion process and made of E-glass and polyester isophthalic resin. The tensile properties of GFRP angle sections as per ASTM standard D638-14 were tested at a loading rate of 1 mm/min in Universal Testing Machine (INSTRON) of capacity 50 kN available at Advanced Material Testing Laboratory in Central Workshop Division. Maximum tensile strength was found to be 487.2 MPa and corresponding ultimate strain was 0.134. Modulus of elasticity in the major direction was found to be 8897 MPa. Figure 3a,b show the test setup and the stress strain curve of GFRP coupons respectively. Volume fraction of fiber was determined as per ASTM D3171-99(04). The fiber volume content of GFRP specimen was found to be 66.67%.

**Figure 4:**
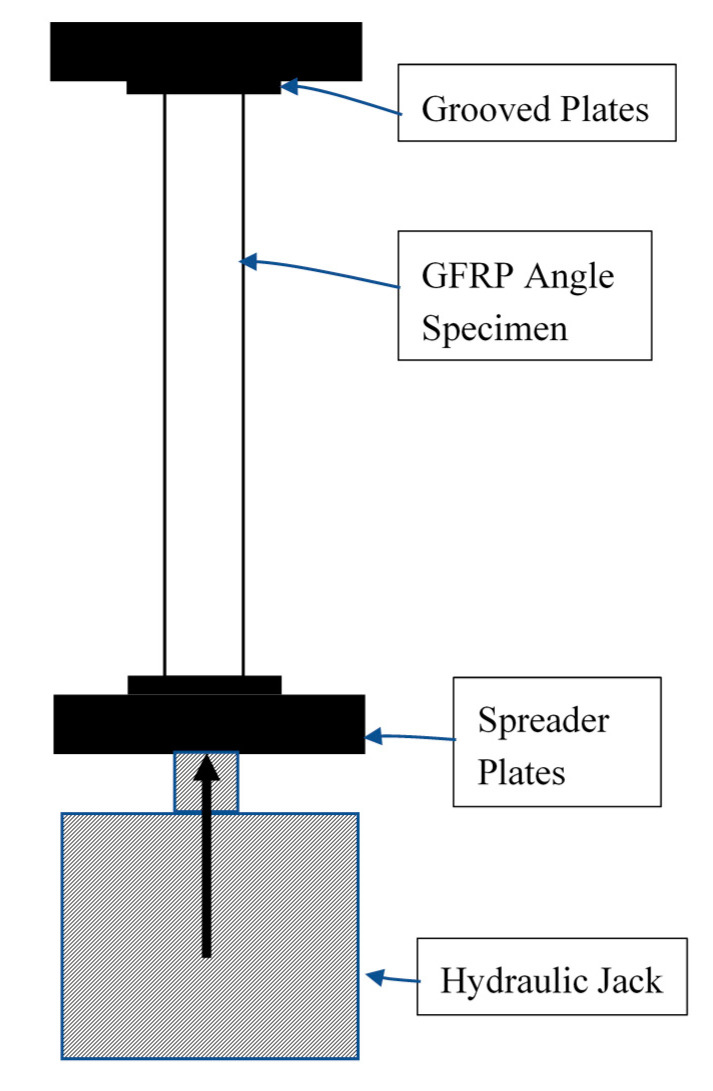
Experimental loading setup.

Geometric properties of the selected angle sections are shown in Table 2. GFRP angle sections were tested under axial load using hydraulic jack of capacity 10 t. Spreader plates were kept at the ends to apply vertical load uniformly over the cross-section. Grooved plates were placed at the extremity of the GFRP angle sections to prevent lateral motion at the ends. Experimental setup is illustrated in Figure 4. The GFRP angle sections were tested up to failure. Axial load, axial deformation, and strains were recorded.

## 4. Results and Discussions

### 4.1. Experimental Results

All the GFRP angle sections were tested up to failure. Strength of the GFRP angle sections is tabulated in Table 3.

#### Failure of GFRP Angle Sections

**Figure 5:**
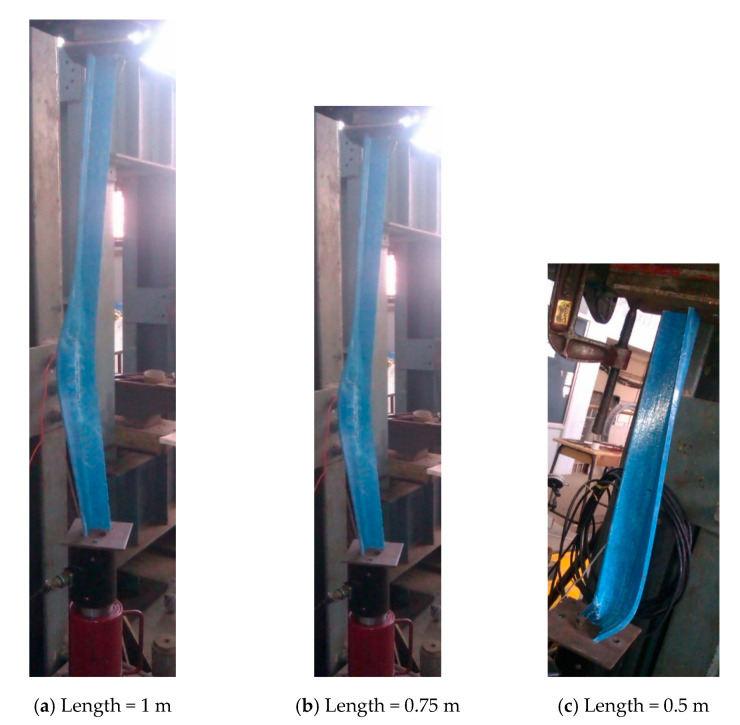
Buckling behavior of GFRP angle sections 50 mm × 50 mm × 6 mm.

**Figure 6:**
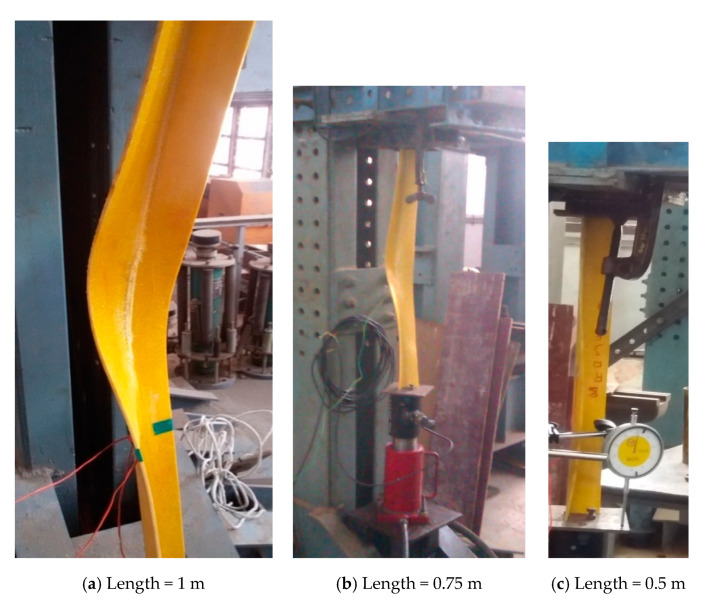
Buckling behavior of GFRP angle sections 50 mm × 50 mm × 4 mm.

GFRP angles of size 50 × 50 × 6 mm and lengths 1000 mm and 750 mm exhibited behavior of opening of the flanges near the mid-height, twisting of the cross-section, and overall bending which is shown in Figure 5a,b respectively. The failure of the specimens was by rupture of the fabric at the flange junction. GFRP angle of size 50 × 50 × 6 mm and length 500 mm exhibited behavior of closing of the flanges near the support and slight twisting. No marked bending of the specimen was observed. The failure was by rupture of fibers in one of the flanges Figure 5c. Behavior of GFRP angle of size 50 × 50 × 4 mm was similar to that of GFRP angles 50 × 50 × 6 mm. GFRP angle of size 50 × 50 × 4 mm and length 1000 mm and 750 mm failed by rupture of the fabric at the flange junction which is shown in Figure 6a,b respectively. GFRP angles 50 × 50 × 4 mm of length 500 mm failed by rupture of the fibers near the support as shown in Figure 6c.

Specimens failed by rupture of GFRP and no marked delamination were observed. All specimens gave warning before failure in the form of noise. The failed specimens returned back to their original geometry on removal of load.

It is observed that the though the modes of failure is similar for sections 50 mm × 50 mm × 6 mm (*b*/*t* = 8.33) and 50 mm × 50 mm × 4 mm (*b*/*t* = 12.5), the ultimate strength is greatly reduced. Ultimate load reduces by about 60% when the b/t ratio is increased from 8.33 to 12.5.

### 4.2. Finite Element Analysis

The tested GFRP angle sections were modelled in ANSYS. A linear elastic orthotropic constitutive law has been adopted for the pultruded FRP material. The material properties based on tensile test and based on properties provided by the manufacturer were input to the model. “SHELL181” quadrilateral element was used to model the GFRP angle sections. “SHELL 181” is capable of large rotation capabilities. Automatic mapped meshing was applied to mesh the geometry. Simply supported boundary conditions were ensured at the extremity by restraining the translational motions. The predominant mode of failure observed during experimental studies was buckling. Hence, buckling analysis using block Lanczos algorithm in ANSYS software was the focus of the simulation. Failure criteria were not adopted in the analysis, in order to give importance to the study of global aspects of the member behavior.

**Figure 7:**
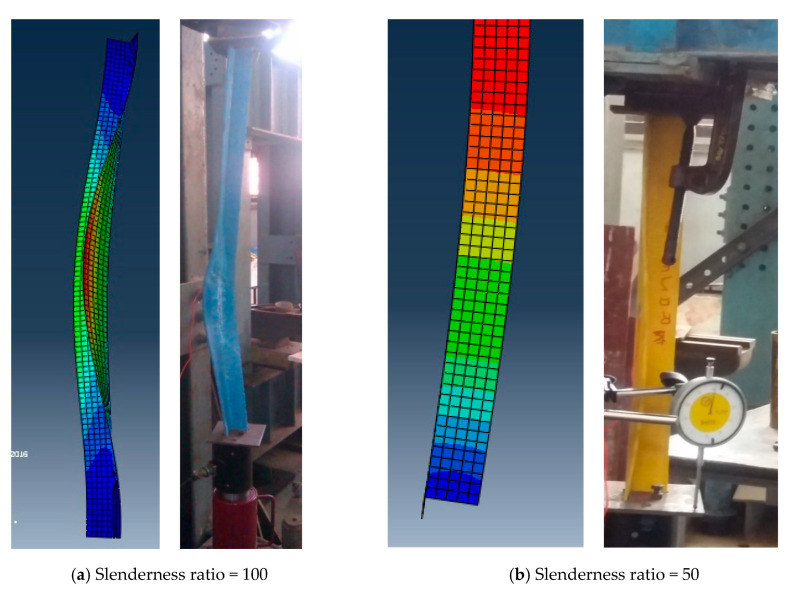
Buckling pattern—Experiment and Finite Element Analysis (FEA).

The critical buckling loads and associated mode shapes were obtained from linear buckling analysis. A comparison of the buckling load and buckling pattern with that of the experimental results were made. Table 3 shows the comparison of buckling loads between finite element analysis and experiments. It was observed that the FE results vary from the experimental results by 5–6%. Figure 7 shows the comparison of buckling pattern between finite element analysis and experiments for slenderness ratios 50 and 100. It is observed that the numerical results based on Finite Element Analysis (FEA) are in good agreement with the experimental results.

### 4.3. Effect of Width-To-Thickness Ratio

It was observed from the experimental study that the thickness of the section and length of the member influences the buckling load. Hence, a parametric study is done varying the width to thickness ratio and slenderness ratio of the angle section.

The width of the section was kept constant and the thickness of the section was varied from 3 mm to 10 mm corresponding to width-to-thickness ratio (*b*/*t*) of 16.67 to 5 [Table 4]. The length of the section was also varied from 500 mm to 1000 mm corresponding to the slenderness ratios of about 50 to 100.

**Figure 8:**
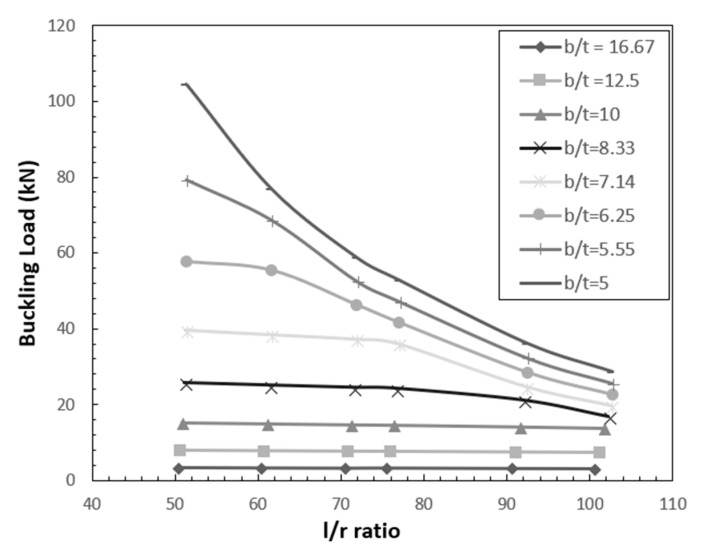
Effect of slenderness ratio and width-to-thickness ratio on buckling load.

Figure 8 shows the variation of buckling load with slenderness ratio for various width-to-thickness ratio. It is observed from Figure 8 that the buckling load decreases with increase in slenderness ratio. The rate of decrease of buckling load increases with the decrease in width-to-thickness ratio (*b*/*t*) ratio.

**Figure 9:**
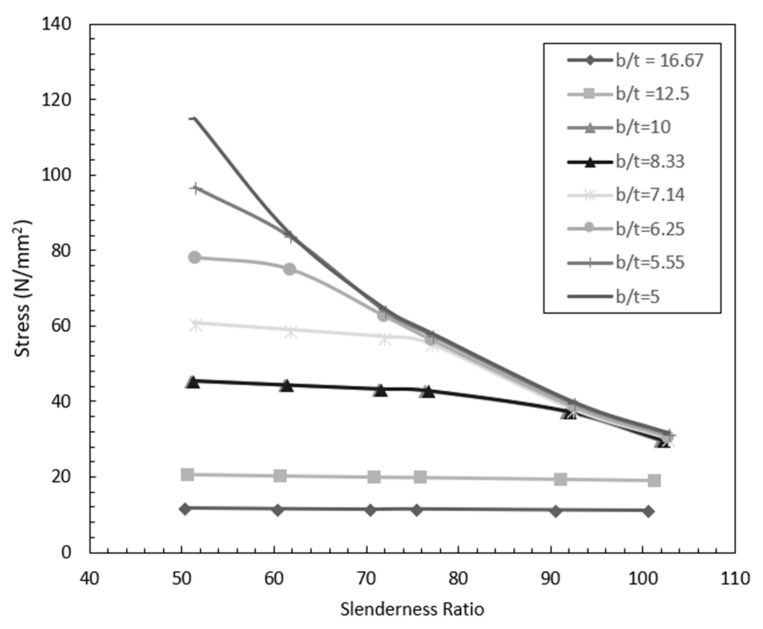
Variation of stress at buckling load with slenderness ratio and width-to-thickness ratio.

**Figure 10:**
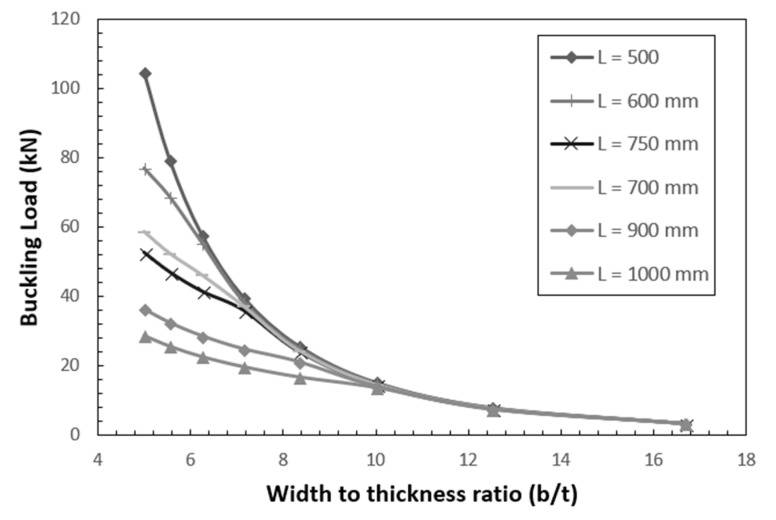
Effect of width-to-thickness ratio on buckling load.

The stress at buckling load was calculated from the buckling load and the variation of stress with slenderness ratio for various width-to-thickness ratio is plotted in Figure 9. It is observed that the buckling load reduces to up to 10% when the width-to-thickness (*b*/*t*) varies from 5 to 16.7. Figure 10 shows the variation of buckling load with width-to-thickness ratio for various slenderness ratios. It is observed that beyond a width-to-thickness ratio of 10, the buckling load of the section is independent of the slenderness ratio.

## 5. Conclusions

In this paper, an experimental and numerical study is carried out on the buckling response of GFRP angle sections of varying thickness and length to bring out the influence of these geometric parameters on the buckling strength and buckling mode. The study involved testing of GFRP angle columns under concentric axial load and development of Finite Element Model (FEM) using ANSYS.

Based on the study on GFRP angle sections under concentric axial compression, the following conclusions are made:

- Specimens of length 750 mm and 1000 mm buckled in overall flexural buckling mode with opening of the flanges. The failure was by rupture of fibers at the junction of the two flanges at mid height.
- Specimens of length 500 mm failed by torsional buckling with closing of the flanges near the loading end. The failure was by rupture of fibers in one of the flanges.
- Based on experimental study, it is observed that an increase in *b*/*t* ratio from 8.3 to 12.5 decreases the load carrying capacity by about 60%.
- Developed finite element model compares well with the experimental study.
- Based on the parametric study, it is observed that the buckling strength decreases with increase in slenderness ratio and width-to-thickness ratio.
- The influence of width-to-thickness ratio is more at lower slenderness ratios. However the study was limited to GFRP angles in the slenderness range of 50 to 100. Future studies can include slenderness ratios beyond these values to have a better understanding of the behavior.

## Figures and Tables

**Table 1:** Ultimate strength of glass fiber reinforced polymers (GFRP) angle sections.

S.No.	Size of GFRP Angle Section	Length (mm)	Longitudinal Modulus of Elasticity (MPa)	*b*/*t*	*l*/*r*	Buckling Load (kN)	
						Experiment	FEA
1	102 × 102 × 12.5	152	50.50	8	79	56 kN	64.4 kN

**Table 2:** Geometry of the glass fiber reinforced polymer (GFRP) angle sections.

FRP Angle Section (mm)	Section Area (mm^2^)	Centre of Gravity C_xx,_ C_yy_ (mm)	Radius of Gyration (mm)			Moment of Inertia ×10^4^ (mm^4^)			
			r_xx_, r_yy_	r_uu_	r_vv_	I_xx_, I_yy_	I_uu_	I_vv_	I_xy_
50 × 50 × 4	384	13.9	15.5	19.5	9.9	9.26	14.7	3.8	5.5
50 × 50 × 6	564	14.7	15.2	19.2	9.8	13.1	20.8	5.4	7.7

**Table 3:** Ultimate strength of GFRP angle sections.

S.No.	Size of GFRP Angle Section	Length (mm)	Slenderness Ratio	Ultimate Strength (kN)		P_FEA_/P_EXP_
				Experiment (P_EXP_)	FEA (P_FEA_)	
1	50 × 50 × 4 mm	500	50.50	11.56	13.92	1.204
2	50 × 50 × 4 mm	750	75.75	7.85	8.31	1.059
3	50 × 50 × 6 mm	500	51.00	27.00	28.83	1.068
4	50 × 50 × 6 mm	750	76.50	21.56	22.68	1.052
5	50 × 50 × 6 mm	1000	102.00	18.15	19.92	1.098
Mean						1.096
Standard Deviation						0.0563

**Table 4:** Size of angle sections considered for parametric study.

Size of the GFRP Angle Section (mm)	Width-To-Thickness Ratio (*b*/*t*)	Area of Cross-Section (mm^2^)	Least Radius of Gyration (mm)
50 × 50 × 3	16.7	291	9.95
50 × 50 × 4	12.5	384	9.88
50 × 50 × 5	10.0	475	9.83
50 × 50 × 6	8.3	564	9.79
50 × 50 × 7	7.1	651	9.76
50 × 50 × 8	6.3	736	9.74
50 × 50 × 9	5.6	819	9.73
50 × 50 × 10	5.0	900	9.73
